# Mitochondrial–Nuclear Epistasis Impacts Fitness and Mitochondrial Physiology of Interpopulation *Caenorhabditis briggsae* Hybrids

**DOI:** 10.1534/g3.115.022970

**Published:** 2015-11-17

**Authors:** Chih-Chiun Chang, Joel Rodriguez, Joseph Ross

**Affiliations:** Department of Biology, California State University, Fresno, California, 93740

**Keywords:** mitonuclear, Dobzhansky-Muller incompatibility, speciation, heteroplasmy, dysfunction

## Abstract

In order to identify the earliest genetic changes that precipitate species formation, it is useful to study genetic incompatibilities that cause only mild dysfunction when incompatible alleles are combined in an interpopulation hybrid. Such hybridization within the nematode species *Caenorhabditis briggsae* has been suggested to result in selection against certain combinations of nuclear and mitochondrial alleles, raising the possibility that mitochondrial–nuclear (mitonuclear) epistasis reduces hybrid fitness. To test this hypothesis, cytoplasmic–nuclear hybrids (cybrids) were created to purposefully disrupt any epistatic interactions. Experimental analysis of the cybrids suggests that mitonuclear discord can result in decreased fecundity, increased lipid content, and increased mitochondrial reactive oxygen species levels. Many of these effects were asymmetric with respect to cross direction, as expected if cytoplasmic–nuclear Dobzhansky-Muller incompatibilities exist. One such effect is consistent with the interpretation that disrupting coevolved mitochondrial and nuclear loci impacts mitochondrial function and organismal fitness. These findings enhance efforts to study the genesis, identity, and maintenance of genetic incompatibilities that precipitate the speciation process.

The formation of species is generally thought to depend on a period of reduced mating and gene flow between two populations (Coyne and Orr 2004). Neutral genetic divergence can then arise and be fixed in each population. Any secondary contact and hybridization between the populations could recombine those population-specific alleles, some of which might have negative epistatic interactions that reduce the fitness of the resulting hybrid (Presgraves 2010). Ultimately, the accumulation of such Dobzhansky-Muller incompatibilities (DMIs, Dobzhansky 1936; Muller 1939; Muller 1940) leads to the formation of biological species (Mayr 1942) once hybrid fitness is altogether eliminated.

A second genetic mechanism that can produce unfit hybrids is the separation of coevolved sets of alleles, often evident as outbreeding depression in highly selfing organisms such as Caenorhabditis nematodes like *C. elegans* (Dolgin *et al.* 2007) and in *Arabidopsis thaliana* (Oakley *et al.* 2015). Thus, the extent to which neutral (DMI accumulation) and selective (coevolution) genetic processes contribute to the formation of species remains unclear.

Identifying the genetic basis of hybrid dysfunction, and thus speciation, has been challenging, in part because genetic divergence will continue to occur after hybrid fitness is eliminated, and incompatibilities are predicted to accumulate at an increasing rate, the “snowball effect” (Orr 1995). It is often difficult to determine the temporal order in which incompatible alleles arose, and then which alleles were responsible for causing speciation as opposed to reinforcing it (Orr 1995; Maheshwari and Barbash 2011), although efforts using a phylogenetic approach have seen success, *e.g.*, Sherman *et al.* (2014).

After identifying loci involved in hybrid dysfunction, more work is then necessary to understand the cellular and molecular mechanisms producing deleterious phenotypic effects in the hybrid (Presgraves 2010; Cutter 2012). Thus, it should not be surprising that many of the best-described incompatibilities exist in model organisms with extensive molecular and genetic toolkits, like fruit flies, yeast, Arabidopsis, and mice (reviewed in (Maheshwari and Barbash 2011)). However, it has also been argued that a broader range of organisms should be used for studying the genetic basis of speciation, particularly in the case of genomic conflict (Johnson 2010).

One form of conflict that has recently been the focus of a number of studies in a wide variety of taxa occurs between the mitochondrial and nuclear genomes. Although mitochondria play a vital role in fitness, their genomes do not encode all of the genes whose protein products are deployed in electron transport chain (ETC) complexes, and are essential for oxidative phosphorylation (reviewed in Rand *et al.* 2004). Instead, many mitochondrial proteins are encoded by genes in the nuclear genome. Because most of the ETC complexes comprise multiple proteins encoded by both genomes, this establishes an opportunity for genomic coevolution. If mitochondrial and nuclear genomes have coevolved to function efficiently together, then mitochondrial function could be reduced in hybrids when hybridization disrupts those coevolved gene complexes (Johnson 2010). At the same time, mitochondrial and nuclear loci are also susceptible to the neutral accumulation of DMIs.

Although the potential involvement of cytoplasmic factors in speciation is an old idea (reviewed in (Orr 1996)), many recent studies have rekindled interest in the notion that mitochondrial genomes might play a major role in speciation (Gershoni *et al.* 2009; Burton and Barreto 2012). Cases of mitonuclear epistasis are useful for studying the genetic basis of hybrid dysfunction, and thus potentially speciation. Identifying hybrids in which negative mitonuclear epistatic interactions cause mild hybrid dysfunction would also potentially avoid issues with the snowball effect (Presgraves 2010).

The empirical identification of such cases can be sporadic. Recent cases often initially identified the potential for mitonuclear epistasis by observing marker transmission ratio distortion (MTRD) specific to one cross direction when working with hybrid crosses, *e.g.*, (McDaniel *et al.* 2007; Corbett-Detig *et al.* 2013; Leppala *et al.* 2013). Our previous work in interpopulation (AF16-HK104) hybrids of the nematode *Caenorhabditis briggsae* also revealed patterns of MTRD that depended on cross direction, suggesting the possibility of mitonuclear epistasis occurring in *C. briggsae* populations (Ross *et al.* 2011). AF16 [a tropical clade strain from India (Fodor *et al.* 1983)] and HK104 (a temperate clade strain from Japan) are two wild isolate populations used routinely in *C. briggsae* genetics. As a primarily selfing species, genetic variants might fix quickly within *C. briggsae* populations, and so strains like AF16 and HK104 tend to be almost or completely homozygous throughout the nuclear genome. These two populations exhibit substantial genetic divergence, with nucleotide substitutions occurring on average every 163 bp (Koboldt *et al.* 2010), and also show some evidence of mild hybrid dysfunction, including an increase in hybrid embryonic lethality and hybrid development rate (Dolgin *et al.* 2008; Ross *et al.* 2011; Baird and Stonesifer 2012). Thus, the combination in *C. briggsae* of population structure (Thomas *et al.* 2015), interpopulation genetic divergence, and evidence of potential mitonuclear epistasis, produces strong potential for identifying the genetic basis of intraspecies hybrid dysfunction. Such work in *C. briggsae* also benefits from its rich suite of genetic and molecular tools (Baird and Chamberlin 2006; Gupta *et al.* 2007; Koboldt *et al.* 2010; Zhao *et al.* 2010).

The first step toward identifying the genetic basis of AF16-HK104 hybrid dysfunction is to determine whether mitonuclear epistasis exists. Deleterious effects of hybridization on fitness resulting from mitonuclear epistatic interactions have been recently described in a number of species. Together, these studies have established requirements for demonstrating that mitonuclear epistasis causes interpopulation hybrid dysfunction (*e.g.*, Breeuwer and Werren 1995; Tiffin *et al.* 2001; Levin 2003; Sackton *et al.* 2003; Bolnick and Near 2005; Zeyl *et al.* 2005; Ellison and Burton 2006; Fishman and Willis 2006; Dowling *et al.* 2007; Ellison and Burton 2008a, 2008b; Ellison *et al.* 2008; Lee *et al.* 2008; Niehuis *et al.* 2008; Gagnaire *et al.* 2012; Gibson *et al.* 2013; Hoekstra *et al.* 2013; Meiklejohn *et al.* 2013; Gershoni *et al.* 2014).

First, genetic variation must exist between the nuclear genomes and the mitochondrial genomes of the two populations. Second, production of cytoplasmic–nuclear hybrids (cybrids) is essential. A cybrid combines the nuclear genome of one population with the mitochondrial genome of the other. If any individual genotype will elicit dysfunction because of the separation of coevolved mitochondrial and nuclear alleles, this is the one. In the presence of negative mitonuclear epistasis that elicits hybrid dysfunction, cybrids are expected to exhibit reduced mitochondrial function and reduced fitness compared to the parental strains. Any phenotypic differences between a cybrid and its paternal parental population (nuclear genome donor) can be attributed to having replaced one mitotype with the other. This comparison rules out the possibility of confounding nuclear–nuclear epistatic interactions that might exist between the parental strains.

In the present study, we experimentally tested the hypothesis that the AF16 and HK104 populations contain mitochondrial and nuclear alleles that negatively interact to produce dysfunctional hybrids. The presence of asymmetric effects of cybridization suggests the accumulation of mitochondrial–nuclear DMIs, while an observed symmetrical effect could be due either to the presence of multiple DMIs or to separation of coevolved mitonuclear loci (Turelli and Orr 2000; Turelli and Moyle 2007). Distinguishing these possibilities is a future goal. Together, these cybrid phenotypes suggest that AF16 and HK104 might occupy an early state of speciation. Our findings support the hypothesis that mitonuclear epistasis exists in *C. briggsae* and promote the use of *C. briggsae* to study the genetic basis of hybrid dysfunction and speciation.

## Materials and Methods

### Nematode husbandry

*C. briggsae* AF16 and HK104 strains were initially obtained from the Caenorhabditis Genetics Center. Strain maintenance and storage were performed as described (Stiernagle 2006). Worms were maintained on nematode growth medium (NGM) agar plates, fed the OP50 strain of *Escherichia coli*, and grown at 20.0°. All experiments were performed on young adult individuals (within 24 hr following the L4/adult molt) derived from populations that had been maintained for at least two generations with food *ad libitum*.

### Sequence analysis

The mitochondrial genome sequences of *C. briggsae* wild isolates AF16 and HK104 were obtained from GenBank (accessions AC186293 and EU40779). Comparison of translated mitochondrial coding regions was performed using ClustalW and the PAM250 matrix (Larkin *et al.* 2007).

### Cybrid production

**Figure 1:**
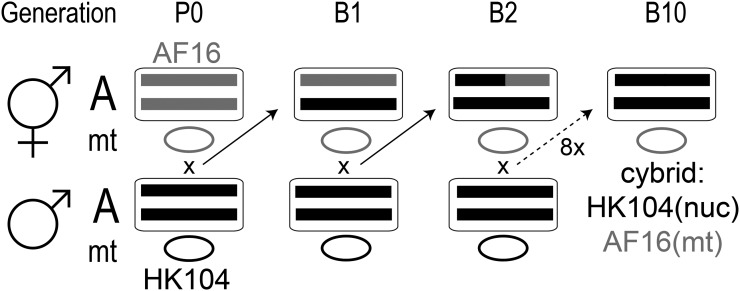
Cross scheme for producing cytoplasmic–nuclear hybrids (cybrids). In the P0 generation, a self-sperm-depleted AF16 (gray alleles) hermaphrodite with diploid nuclear genome (an example autosome pair, A, shown, in the nucleus, rounded rectangle), and mitochondrial genome (a representative circular genome, mt) is crossed to an HK104 (black alleles) male. This cross produces an F1 hybrid individual with an entirely heterozygous nuclear genome, and maternally (hermaphroditically)-inherited mitochondrial haplotype. Backcrossing the self-sperm-depleted F1 hermaphrodite to another male from the P0 paternal strain produces the first backcross (B1) generation, and begins to introgress the paternal (HK104) nuclear genome onto the maternal (AF16) cytoplasm. Ten generations of backcrossing produces B10 cybrids. The nuclear genome should be homozygous for P0 paternal (HK104) alleles, while maternal inheritance of cytoplasm should produce a cybrid with AF16 mitochondria. The cross shown produced cybrid replicates CP131, 132 and 133. The reciprocal cross (P0 HK104 hermaphrodite and AF16 male; serial backcross of hybrid hermaphrodites to AF16 males) produced replicates CP129 and CP130.

*C. briggsae*, like its close relative *C. elegans*, comprises individuals of two sexes: males and self-fertile hermaphrodites (Gupta *et al.* 2007). Cytoplasmic–nuclear hybrids (cybrids) were created using the cross scheme shown in Figure 1. Cybrids were produced in both cross directions, using either one HK104 self-sperm-depleted hermaphrodite mated to AF16 males, or an AF16 self-sperm-depleted hermaphrodite mated to HK104 males in the P0 generation. A single F1 hybrid hermaphrodite for each line was then self-sperm-depleted, and backcrossed to additional males from the same strain as the P0 generation males. Replicate lines for each cross direction were established at the F1 generation and subsequently backcrossed separately through a single sperm-depleted hermaphrodite per generation, with the goal of establishing three replicate cybrid lines per cross direction. However, during the serial backcross, one line established by the P0 cross AF16 male x HK104 hermaphrodite was lost because of absence of oocyte production following self-sperm depletion in an advanced generation of the cross. Thus, two cybrid replicate lines with P0 AF16 fathers and HK104 mothers (strains CP129 and CP130), and three replicates (CP131–CP133) with P0 HK104 fathers and AF16 mothers were produced. From the F1, backcrossing was repeated for nine generations to generate B10 cybrids.

### Genotyping

To establish that the expected genotypes were obtained after the cybrid cross scheme, each line was genotyped at one single nucleotide polymorphism (SNP) per nuclear chromosome and one SNP in the mitochondrial genome, an approach used in the evaluation of *C. briggsae* cybrid genotypes in Hicks *et al.* (2012). Insertion-deletion (indel) markers (Table 1 and Supporting Information, Figure S1) that distinguish AF16 and HK104 alleles were amplified by polymerase chain reaction (PCR) using published primer sequences (Koboldt *et al.* 2010).

**Table 1:** Observed single nucleotide polymorphism (SNP) genotypes of parental and cybrid lines

SNP	Chr	Mbp	AF16	HK104	CP129	CP130	CP131	CP132	CP133
Cb-m26	II	11.867	A	H	A	A	H	H	H
Cb-m154	III	2.166	A	H	A	A	H	H	H
Cb-m205	III	5.649	A	H	A	A	H	H	H
Cb-m172	IV	10.305	A	H	A	A	H	H	H
Cb-m74	IV	15.657	A	H	A	A	H	H	H
Cb-m103	V	11.376	A	H	A	A	H	H	H
Cb-m124	X	12.616	A	H	A	A	H	H	H
cb18178	mt		A	H	H	H	A	H	A

Seven strains (wild isolates AF16 and HK104, and five cybrids: CP129–CP133) were genotyped at the eight SNPs listed. Columns contain the name of each SNP (Koboldt *et al.* 2010; Ross *et al.* 2011), the chromosome (Chr) it is located on [autosomes I, II, III, IV, V; the X chromosome, or the mitochondrial (mt) genome], and its position in Mbp on that chromosome (assembly cb4). The remaining columns list the genotype of each strain at each SNP. For the diploid nuclear loci: homozygous AF16/AF16 alleles (A), homozygous HK104/HK104 alleles (H). No heterozygous genotypes were identified. Because all of the genotyping assays for the mitochondrial locus appeared to reveal homoplasmy, a single allele (A, AF16 homoplasmy; H, HK104 homoplasmy) is listed.

The AF16-HK104 SNP cb18178 was previously determined to be located in the mitochondrial genome (Ross *et al.* 2011). Alignment of the mitochondrial genome sequences of AF16 and HK104 (GenBank accessions AC186293 and EU407793.1) identified that cb18178 acts as a restriction fragment length polymorphism (RFLP) marker, causing differential presence of a *Sex*AI restriction site. PCR amplification from the AF16 and HK104 mitochondrial genomes (mtDNA) is predicted to produce a 798-bp amplicon at cb18178. *Sex*AI should not digest the amplicon from AF16; digest of the HK104 allele is expected to result in 164 and 633 bp products. Forward and reverse PCR primers CACTTACAATGATAGGGTTTAAAATTC and CCATTTCTGCGAAAAAGAAAC were designed to amplify cb18178 from the mitochondrial genomes of both strains. All amplifications occurred in a Bio-Rad T100 thermal cycler using the following cycle sequence: 95° 2 min, 30 cycles of 95° 1 min, 45° 1 min, 72° 2 min, followed by a final 72° 5 min extension, and subsequently stored at 4°. After amplification, cb18178 amplicons were digested using restriction endonuclease *Sex*AI (New England Biolabs) using the manufacturer’s recommended protocol. All amplicons were separated electrophoretically on 1% agarose gels in 1x TRIS-acetate EDTA buffer containing 2 µg/mL ethidium bromide. Micrographs of gels were obtained using an AlphaImager HP system (Alpha Innotech).

### Mitochondrial heteroplasmy genotyping

The presence and relative amount of mitochondrial heteroplasmy for a recurring deletion in the *ND5* gene (*ND5*∆) was assessed in purified genomic DNA extracts by PCR as above with the following change: the primers used to amplify the *ND5* locus were CbMt_1F and 58R (Howe and Denver 2008). Quantification of the relative amounts of intact and ND5∆ amplicons was performed by micrograph densitometry of the ethidium bromide-stained agarose gel using ImageJ (Rasband 1997–2015). The percent of *ND5*∆ heteroplasmy for each sample was calculated from the ratio of the integrated pixel density values of the *ND5*∆ amplicon to the sum of the *ND5*∆ and intact amplicon integrated density values.

### Cybrid phenotyping and statistical comparison

As is convention in the literature for analysis of cybrid phenotypes (*e.g.*, Sackton *et al.* 2003; Ellison and Burton 2006; Ellison *et al.* 2008; Hicks *et al.* 2012), we first combined data from independent experimental and biological replicates and then compared each cybrid cross direction to its paternal parental population. That is, all data from all experimental replicates of both cybrid strains with AF16 nuclear and HK104 mitochondrial genotypes (CP129 and CP130, the biological replicates) were combined and then compared to AF16; all CP131 and CP133 cybrid data (HK104 nuclear and AF16 mitochondrial genotypes) were combined and then compared to HK104. Statistical differences were assessed using two-tailed, unpaired Student’s *t*-test for samples with unequal variances with a significance threshold of *P* = 0.05 after Bonferroni correction.

### Fecundity measurement

To measure the number of viable self-offspring produced by an individual hermaphrodite, animals were isolated onto agar plates as larvae; each animal was moved to a new plate daily from the onset of offspring production until the cessation of self-fertility. Three days after the adult hermaphrodite was removed from a plate, the number of living offspring on that plate was manually tallied.

### Reactive oxygen species measurement

*In vivo* measurement of reactive oxygen species (ROS) levels in individual nematodes was performed as described Dingley *et al.* (2010) with modifications. All work was performed in the darkest conditions possible. Briefly, MitoSOX Red (Invitrogen) was reconstituted to 1 mg/ml in dimethyl sulfoxide (DMSO), and then diluted 3.29-fold in S basal medium; 130.4 µl of this MitoSOX Red solution was added to the OP50 lawn on a 4 ml (35-mm diameter) NGM plate. A DMSO/S basal-only plate was prepared as a negative control. The following day, individual worms from each strain (cybrid as well as parental control) were transferred onto both types of plates, where they ingested OP50 along with either MitoSOX dye or vehicle. After 24 hr, the worms were transferred onto NGM plates with unlabeled OP50 for 1 hr to clear their digestive tracts of labeled bacteria. Worms were then transferred onto 1% agarose pads on microscope slides in 10 µl of 50 mM levamisole in M9 buffer, and a coverslip was added. Differential interference contrast (DIC), and fluorescence micrographs of the anterior of each worm were captured through a 40x PLAN-NEUFLUAR objective on a Zeiss Axioimager.A1 compound microscope using an Axiocam mRm and Axiovision 4.8 software. Zeiss filter set 20 (BP 546/12, FT 560, BP 575-640) was used with an X-cite series 120Q (Excelitas) light source for fluorescence microscopy.

**Figure 2:**
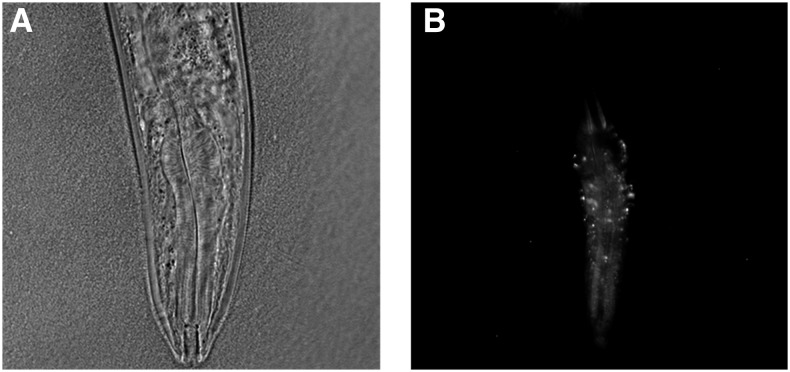
MitoSOX Red fluorescence quantitation in the nematode pharynx. A) DIC micrograph (40x objective) of the anterior of a *C. briggsae* adult individual. B) MitoSOX Red fluorescence overlay in the pharyngeal bulb of the same individual.

MitoSOX Red fluorescence was then quantitated using ImageJ (Rasband 1997–2015). The terminal pharyngeal bulb of each individual was manually encircled in the DIC image of a worm; the integrated pixel density of that region was then obtained from the corresponding fluorescent image (Figure 2). Background autofluorescence was estimated as the average fluorescence of all individuals on the vehicle treatment plate; this value was subtracted from the integrated density measurement of each MitoSOX Red-treated worm. To account for possible instrument drift across multiple days, background-corrected fluorescence values for all strains were normalized so that the individual worm with the highest fluorescence reading in each experimental session had a relative fluorescence measurement of 1.

### Lipid content measurement

*In vivo* measurement of lipid content was performed by staining using the lipophilic fluorescent dye Nile red (Sigma) following van den Ecker *et al.* (2012) with the following modifications. Young adults were transferred to NGM plates that had been prepared by pipetting 0.25 ml Nile red solution (100 ng/ml in acetone) onto the OP50 lawn, and letting the plate incubate for 24 hr. Nematodes ingested the dye together with OP50 for 24 hr and were then moved to unlabeled OP50 on NGM plates for an additional 24 hr to clear the digestive tract of Nile red. Control individuals were treated identically, except that they were only exposed to vehicle. Worms were then mounted and micrographs obtained as above for ROS phenotyping. Fluorescence was quantified as the difference between raw integrated density of whole-body fluorescence of experimental individuals, and background fluorescence measurements from control individuals using ImageJ (Rasband 1997–2015).

### Data availability

Strains CP129–CP133 are available upon request.

## Results

### Mitochondrial genomic variation in C. briggsae

To assess the potential for mitonuclear coevolution in AF16 and HK104, their mitochondrial genome sequences were aligned. Comparison of mitochondria-encoded electron transport genes from AF16 and HK104 (Table 2) reveals that two of the 12 genes analyzed are identical in amino acid sequence in both wild isolates. The other 10 have percent similarities ranging from 99.5 to 97.4. Seven of those 10 have at least one dissimilar amino acid substitution.

**Table 2:** Comparison of amino acid similarity in the protein-coding genes of the AF16 and HK104 mitochondrial genomes

ETC	Gene	S	W	–	%
I	ND6	2	0	1	97.9
I	ND4L	1	0	1	97.4
I	ND1	3	0	1	98.6
V	ATP6	1	0	0	99.5
I	ND2	2	3	0	98.6
III	CTB	1	2	1	98.9
IV	CO3	0	0	0	100.0
I	ND4	3	1	2	98.5
IV	CO1	1	0	2	99.4
IV	CO2	0	0	0	100.0
I	ND3	1	0	0	99.1
I	ND5	2	3	1	98.9

Columns provide: the electron transport chain (ETC) complex to which each gene belongs [ND, NADH-ubiquinone oxidoreductase; CTB, cytochrome B; CO, cytochrome *c* oxidase, nomenclature following (Lemire 2005)], the number of strongly similar substitutions (S, Gonnet PAM250 matrix score > 0.5), weakly similar substitutions (W, score 0–0.5), or dissimilar substitutions (–, negative score), and the protein percent similarity (%). Genes are arranged in order of their position in the *C. briggsae* mitochondrial genome

### Cybrid genotypes

The two cybrid replicate strains with AF16 as the male (nuclear donor) parent and HK104 as the hermaphrodite (mitochondrial donor) parent (CP129 and CP130), and three strains with HK104 as the male parent and AF16 as the hermaphrodite parent (CP131–CP133), were genotyped to confirm the success of the cybrid cross scheme (Table 1 and Figure S1). The two cybrids (CP129 and CP130) whose F1 hybrid ancestor was repeatedly backcrossed to HK104 males were homozygous for HK104 alleles at each nuclear locus tested. The three cybrids (CP131–CP133) whose F1 hybrid ancestor was repeatedly backcrossed to AF16 males were homozygous for AF16 alleles at each nuclear locus tested.

**Figure 3:**
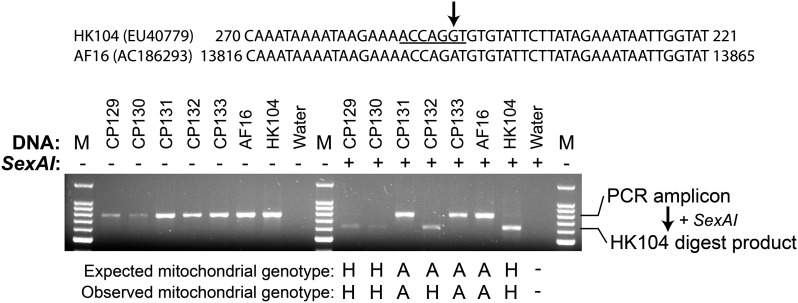
Mitochondrial RFLP genotyping assay. The cb18178 SNP (upper black arrow) eliminates a *Sex*AI restriction endonuclease site (underlined) in the AF16 mitochondrial genome. An initial PCR amplicon of 798 bp should be produced (*Sex*AI –, left half of gel) from either mitochondrial haplotype (AF16 or HK104). Subsequent addition of *Sex*AI (+, right half of gel) should result in digest products of 634 bp (shown) and 164 bp (not shown) only from HK104 alleles. DNA template sources are listed above each lane; M: 100 bp molecular weight marker. Interpretations of observed genotypes are listed underneath the digested lanes (H: HK104 homoplasmy; A: AF16 homoplasmy). Expectations are based on maternal inheritance of mitochondria and the cross design used to produce each cybrid (Figure 1).

Agarose gel electrophoresis of the *Sex*AI-digested cb18178 PCR amplicons from AF16, HK104, and the five cybrid lines revealed the mitochondrial genotypes of the five cybrids (Figure 3). The AF16 amplicon is ∼800 bp in size before digest. Addition of *Sex*AI to these AF16 amplicons results in no digestion product. The HK104 amplicons digest completely, as expected for HK104 alleles. Amplicons from strains CP129, CP130, and CP132 digest to completion; strains CP131 and CP133 do not show any visible evidence of digestion product.

**Figure 4:**
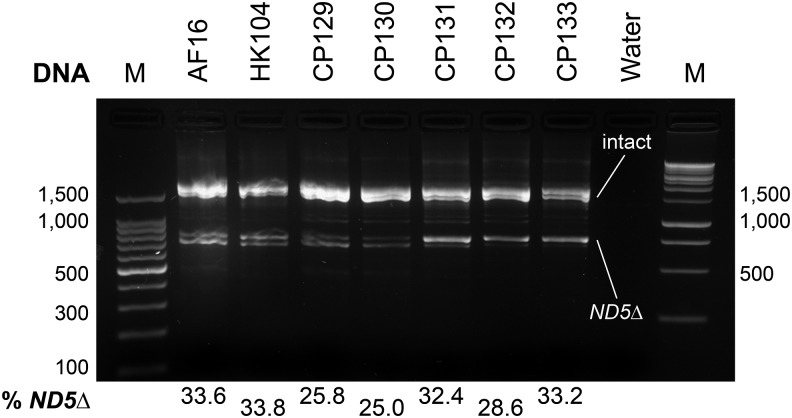
Assessment of mitochondrial heteroplasmy for *ND5***∆**. PCR amplification of the *ND5* locus with primers that flank a commonly occurring deletion (*ND5*∆) produces two amplicons: the shorter deletion-bearing product (∼800 bp) and the intact product (∼1700 bp) (Howe and Denver 2008). The percent of these amplicons represented by *ND5*∆ products is shown beneath the agarose gel image for the parental strains, five cybrids, and a negative (water) control. M: molecular weight ladders (100 bp on left; 1 kbp on right).

Upon amplification of purified genomic DNA from AF16, HK104, and the five cybrid replicates using primers flanking a common heteroplasmic mitochondrial genome deletion (Howe and Denver 2008), amplicons representing intact and ND5∆ mitotypes were generated (Figure 4). The levels of *ND5*∆ in AF16 and HK104 were 33.6% and 33.8%, respectively. ND5∆ heteroplasmy in the cybrids ranged from 25.0% to 33.2%.

Because the mitochondrial genotype of CP132 disagreed with expectation, CP132 was omitted from further experiments.

### Cybrid fecundity

**Figure 5:**
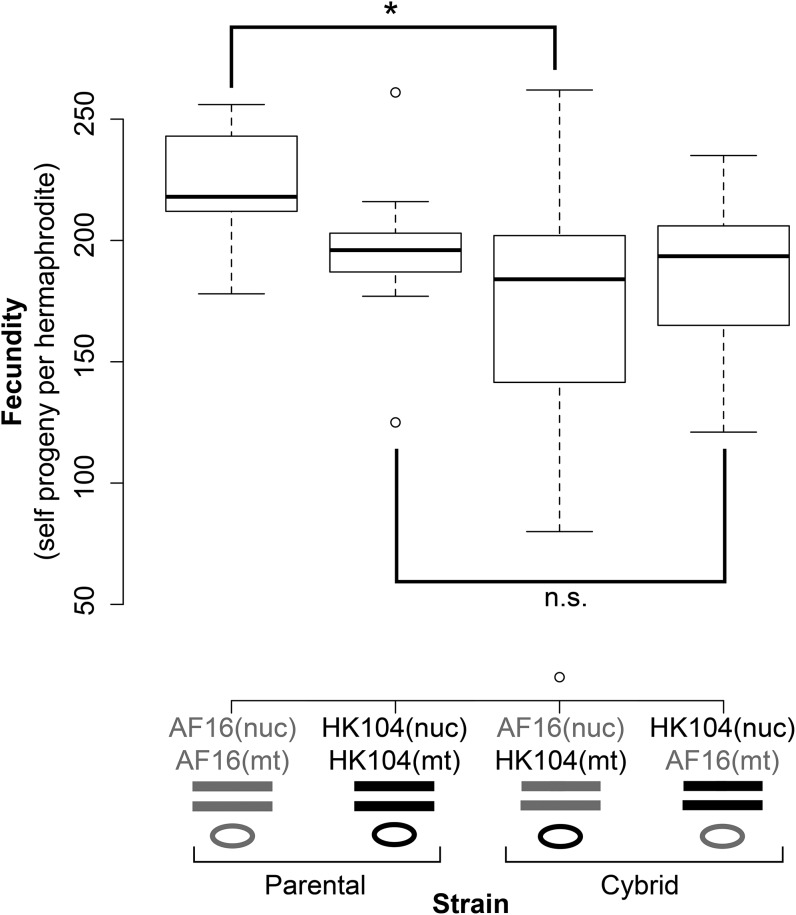
Comparison of parental and cybrid fecundity. Boxplot of the fecundities of the two P0 parental strains (AF16 and HK104) and the cybrids. The cybrids with AF16 nuclear and HK104 mitochondrial genotypes exhibit significantly lower fecundity than AF16 (*P < 0.05, n.s. not significant; Bonferroni-corrected unpaired two-tailed *t*-test).

To identify the effect of genotype on organismal phenotype, the number of hermaphrodite self-progeny surviving to adulthood (self-brood size) was determined for the parental strains AF16 and HK104, and for the cybrid strains CP129 and CP130 (AF16 nuclear and HK104 mitochondrial genotypes), and CP131 and CP133 (HK104 nuclear and AF16 mitochondrial genotypes). Comparison of AF16(nuc); HK104(mt) to the fecundity of wild-type AF16 revealed a significant decrease in cybrid fecundity in this hybrid cross direction (*P* < 0.05) (Figure 5 and Table S1). There was no significant difference between HK104(nuc); AF16(mt) cybrids and HK104.

### Cybrid ROS

**Figure 6:**
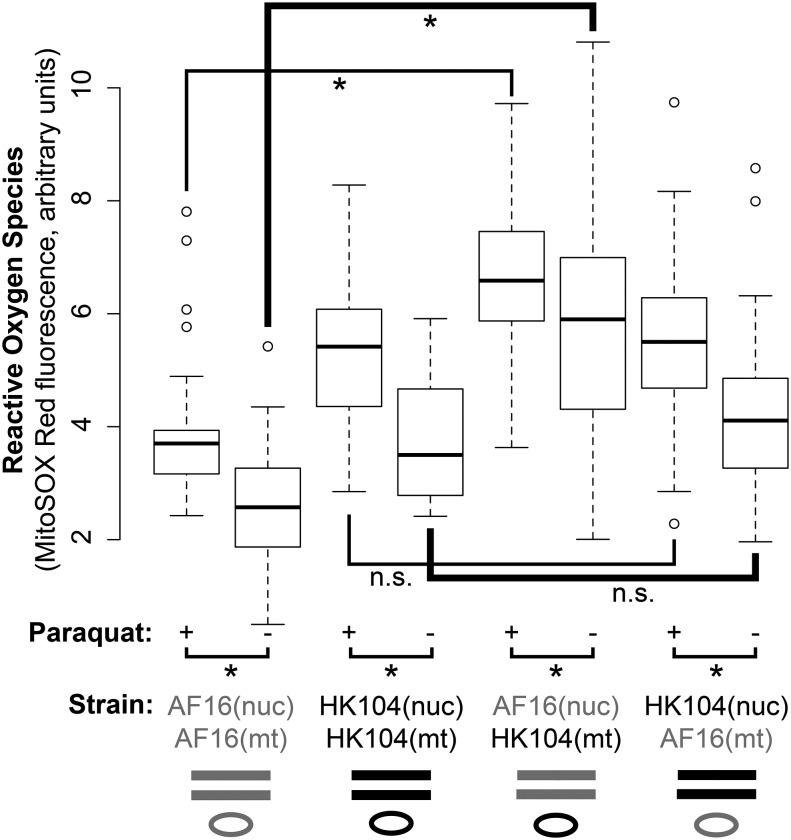
Comparison of parental and cybrid reactive oxygen species (ROS) levels. Boxplot of *in vivo* ROS measured by MitoSOX Red fluorescence. Horizontal brackets within the plot denote four statistical comparisons made between cybrids and their P0 paternal strains (*P < 0.05, n.s. not significant; Bonferroni-corrected unpaired two-tailed *t*-test). The cybrids with AF16 nuclear and HK104 mitochondrial genotypes exhibit significantly higher ROS levels than AF16 both in the absence (–) and the presence (+) of ROS-inducing paraquat. For every strain, the presence of paraquat significantly increases ROS levels (four horizontal brackets beneath the plot). Different thicknesses of the lines denoting statistical comparisons are for clarity.

*In vivo* ROS levels were measured in cybrid and parental strains by quantifying the fluorescence of ingested MitoSOX Red mitochondrial superoxide indicator (Figure 6 and Table S2). AF16(nuc); HK104(mt) cybrid ROS levels are increased compared to AF16, but HK104(nuc); AF16(mt) cybrid ROS levels do not differ from HK104. As a positive control, the addition of paraquat to the nematode medium significantly increased detected ROS levels in every strain. Even in the presence of paraquat, the AF16(nuc); HK104(mt) cybrids maintained a significant increase of *in vivo* ROS compared to AF16, and the HK104(nuc); AF16(mt) cybrids still demonstrated no significant difference from HK104.

### Cybrid lipid content

**Figure 7:**
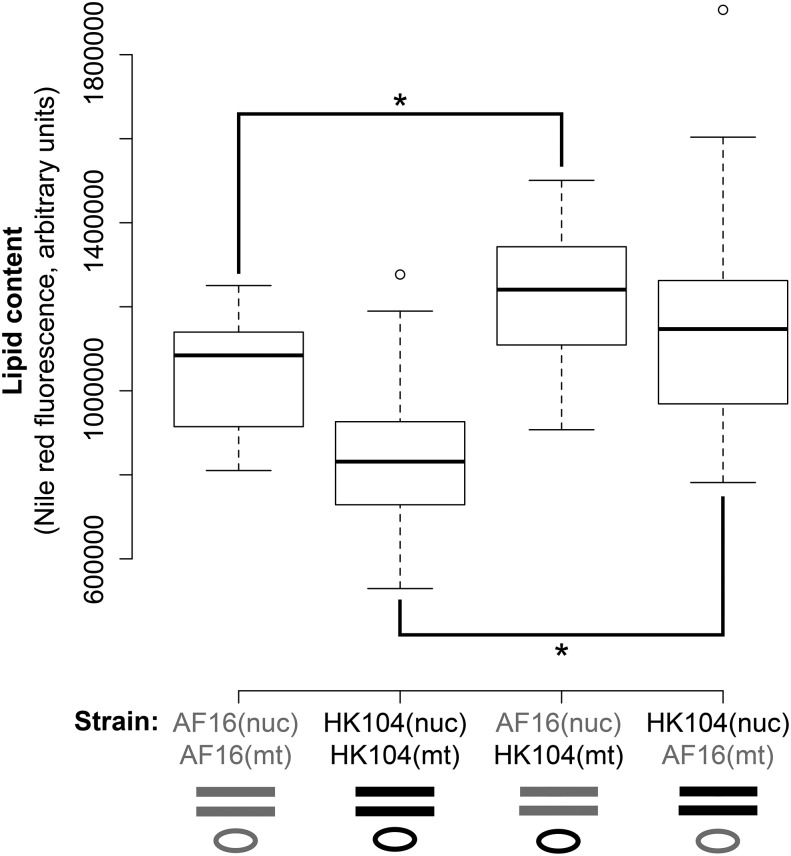
Comparison of parental and cybrid lipid content. Boxplot of whole-animal lipid content measured by Nile red fluorescence. Comparisons between replicate cybrids and the paternal parental strain revealed significant increases in lipid content in cybrids (*P < 0.05; Bonferroni-corrected unpaired two-tailed *t*-test).

Quantification of *in vivo* Nile Red fluorescence in parental and cybrid strains revealed a statistically significant increase in whole-body lipid content in both cybrid cross directions compared to the paternal parental strains (Figure 7 and Table S3).

## Discussion

The possibility of mitonuclear epistasis in *C. briggsae* was first suggested by asymmetric patterns of MTRD in reciprocal hybrid crosses (Ross *et al.* 2011). In hybrids of other species, MTRD specific to one cross direction had previously been reported to result from mitonuclear epistasis (McDaniel *et al.* 2007; Niehuis *et al.* 2008) and to overlap quantitative trait loci for hybrid incompatibility (Moyle and Graham 2006). We therefore asked whether mitonuclear genetic incompatibility has arisen between the *C. briggsae* wild isolates AF16 and HK104. By creating, genotyping, and phenotyping replicate cybrids, we have shown that cybrids have reduced fitness and altered mitochondrial physiology. Thus, *C. briggsae* AF16-HK104 cybrids display the characteristics of negative mitonuclear epistasis.

### Mitochondrial sequence variation

In order to invoke negative mitonuclear epistasis as an explanation for the observed cybrid dysfunction, the parental strains should exhibit genomic divergence. The AF16 and HK104 mitochondrial genomes are 98% identical. In yeast, mitochondrial genomes with >98% sequence identity are unable to substitute effectively for each other (Zeyl *et al.* 2005; Wei *et al.* 2007). In Tigriopus copepods, hybrids that suffer from mitochondrial dysfunction have mitochondrial genome-encoded cytochrome oxidase I sequences whose divergence exceeds 15% (Ellison and Burton 2006). In *Drosophila simulans*, <0.1% divergence in mitochondrial genomes is sufficient to produce negative mitonuclear epistasis (Meiklejohn *et al.* 2013). It has also been suggested, in response to the advent of three-parent fertilization to avoid maternal transmission of pathogenic mitochondrial mutations (Callaway 2014), that the degree of genetic variation in human genomes puts individuals engineered by mitochondrial replacement at risk for negative mitonuclear incompatibilities (Morrow *et al.* 2015). Thus, the amount of mitochondrial genome divergence between AF16 and HK104 falls in the range of divergence that has been shown to sustain negative mitonuclear epistasis.

We also specifically assessed whether any of the sequence divergence in AF16 and HK104 mitochondria might cause nonsynonymous changes to proteins. The presence of nonsynonymous substitutions in mitochondrial genomes had been previously noted for temperate strains (Howe and Denver 2008), and the mitochondrial genomes of some populations sustain large heteroplasmic deletions that are expected to negatively impact fitness (Howe and Denver 2008; Howe *et al.* 2010; Estes *et al.* 2011). We found that seven of the 12 mitochondrial-encoded electron transport chain genes contain nonsynonymous substitutions (Table 2) with divergence levels in accord with the genome-wide value. Thus, the mitochondrial protein-coding divergence could provide a substrate for negative mitonuclear epistasis. However, no molecular evolutionary data yet support the hypothesis of positive selection in *C. briggsae* mitochondrial-encoded ETC components (Howe and Denver 2008); such a finding would suggest the potential for mitonuclear coevolution. No such analysis has yet been performed for the *C. briggsae* nuclear-encoded mitochondrial-acting genes (Hicks *et al.* 2012).

Beyond oxidative phosphorylation, many other opportunities exist for epistasis between the mitochondrial and nuclear genomes. Mitochondria integrate signals controlling lipid metabolism, apoptosis, development rate, and cell cycle control. Thus, although much research focus has been placed on understanding mitonuclear interactions impacting oxidative phosphorylation, other forms of mitonuclear epistasis could impact fitness. Not only might epistatic interactions involve numerous candidate nuclear loci beyond the ETC genes, but also they potentially involve variation in noncoding regions (*e.g.*, Nam and Kang 2001; Karlok *et al.* 2002; Ellison and Burton 2008a; Meiklejohn *et al.* 2013). Likewise, mitochondrial-nuclear epistasis between the human X-linked gene *KAL1* and mitochondrial tRNA^cys^ results in Kallmann syndrome (Wang *et al.* 2015). Future efforts in the field should incorporate a wider perspective on the potential genetic basis of mitochondrial-nuclear epistasis.

### Cybrid production and genotyping

To test the hypothesis that mitonuclear epistasis has a deleterious effect on AF16-HK104 hybrids, we created reciprocal and replicate cybrids. Serial backcrosses of F1 hybrids to males from the P0 paternal population were employed to eventually return the nuclear genotype to homozygosity for the P0 paternal alleles while maintaining the P0 maternal mitochondrial haplotype by maternal cytoplasmic inheritance (Figure 1), which occurs in Caenorhabditis (Lemire 2005) and which has also been shown to be true for serial backcrosses in mouse, for example Gyllensten *et al.* (1985). Table 1 summarizes the cybrid genotype data. For each nuclear locus tested, CP129 and CP130 were homozygous for AF16 alleles and CP131–CP133 were homozygous for HK104 alleles, as expected.

### Mitochondrial inheritance in C. briggsae hybrids

We next identified the mitochondrial haplotypes of the cybrid lines using an RFLP genotyping assay (Figure 3). Absence of digest product in the AF16 lane, and complete digest of amplicons in the HK104 lane, indicates that both parental strains are homoplasmic for their respective mitochondrial haplotypes, eliminating the possibility of heteroplasmy at the onset of our crosses (Howe and Denver 2008; Estes *et al.* 2011).

For all but one of the strains, the mitochondrial genotype was consistent with the cybrid cross design. All CP132 amplicons are digested in the presence of *Sex*AI, indicating that CP132 is homoplasmic for HK104 mitochondria. Similar mitotype results were also obtained for CP132 using a different RFLP assay (not shown). This result is remarkable, considering that no HK104 hermaphrodites (and thus no maternal source of HK104 mitotypes) were employed in the ten generations of crossing used to produce CP132.

Two notable implications of this finding are i) that paternal mitochondrial transmission occurred, and ii) that the resulting heteroplasmy for AF16 and HK104 mitochondria that must initially have existed following paternal transmission was rapidly and completely resolved in favor of transmission of HK104 mitochondria, producing a cybrid line apparently homoplasmous for HK104 mitochondria within 10 generations.

It is possible that AF16 mitochondrial genomes do remain in low relative copy number that is not detectable by eye (Figure 3). Comparison of qualitative PCR (qPCR) and PCR results for *C. briggsae* mitochondria suggests that a conservative estimate of the limit of detection of heteroplasmy by PCR is at least as low as 5% (Howe and Denver 2008). Further, because the cb18178 RFLP assay digests only HK104 mitochondrial amplicons, incomplete digest, absence or inactivity of restriction endonuclease, or other related technical artifacts cannot explain this unexpected result. To ensure that amplification by the cb18178 primer is not biased toward one mitotype, the primer sequences were aligned to the AF16 and HK104 mitochondrial genome assemblies. The primers are identical to the AF16 mitotype; one SNP exists between the forward primer and the HK104 mitotype. As such, it is also unlikely that biased amplification of HK104 mitotypes explains the CP132 RFLP data.

A similar result of paternal mitochondrial transmission after hybridization of different *C. briggsae* parental strains has also been reported (Hicks *et al.* 2012), raising the distinct possibility that some male mitochondrial transmission occurs at fertilization in *C. briggsae* hybrids. This interpretation is in accordance with findings from *C. elegans* (Al Rawi *et al.* 2011; Sato and Sato 2011). In sum, we conclude it is unlikely that the anomalous mitotype of CP132 is due to experimental artifact. Because of the discrepancy between the expected and observed mitotype of CP132, it was subsequently omitted from all phenotypic analyses reported here.

### Cybrid ROS production

If existing mitochondrial nucleotide variation in AF16 negatively interacts with nuclear variants in HK104, or *vice versa*, then the production of cybrids might result in ETC dysfunction. ROS are produced as a byproduct of oxidative phosphorylation (Nohl and Hegner 1978; Nohl 1986), and tend to increase because of inefficient electron transport resulting from mitochondrial DNA damage. Mitochondrial dysfunction resulting from negative mitonuclear epistasis might be evident as an increase in ROS production by cybrids. Many *C. elegans* ETC mutants produce increased ROS levels (Dingley *et al.* 2010), although ROS levels in *C. briggsae* populations have not found to correlate linearly with the population frequencies of specific mitochondrial genetic variants (Estes *et al.* 2011; Hicks *et al.* 2012).

Our cybrid phenotyping revealed that combining HK104 mitochondria with the AF16 nuclear genome, but not *vice versa*, increases *in vivo* mitochondrial ROS levels (Figure 6). The asymmetry seen in the ROS level phenotype suggests the presence of a mitonuclear DMI between the HK104 mitochondrial and AF16 nuclear genomes. These results reveal the impact of mitochondrial genotype on mitochondrial phenotype: replacing the mitochondrial genome with another mitotype from a different population results in an increase in ROS levels. This interpretation is consistent with a prior conclusion that a genetic effect on ROS production exists in cybrids between AF16 and a third strain, HK105 (Hicks *et al.* 2012). The mitochondrial genetic effect on ROS production we observe in cybrids is consistent across conditions (both in the presence and absence of paraquat, Figure 6). However, it should be noted that we are yet unable to distinguish whether an increase in ROS level might be due to decreased efficiency of ROS scavenging (*e.g.*, by superoxide dismutase) and/or increased production of ROS (Turrens 2003; Estes *et al.* 2011).

### Cybrid lipid content

Studies in Caenorhabditis and in humans with mitochondrial myopathies have suggested the possibility that altered mitochondrial function impacts lipid storage (Bonilla and Prelle 1987; Kimura *et al.* 1997; Chow and Thorburn 2000; Mak *et al.* 2006; Mailloux *et al.* 2007; Jones *et al.* 2009; Soukas *et al.* 2009; Liu *et al.* 2014). Thus, we stained parental strains and cybrids with Nile red to quantitate and compare whole-individual lipid content. Both cybrid cross directions resulted in a statistically significant increase in lipid content (Figure 7), further supporting the interpretation that negative epistatic interactions in cybrids impact mitochondrial function.

The symmetry of the effect of mitotype on cybrid lipid content suggests that AF16 mitochondria are incompatible with an HK104 nuclear allele; HK104 mitochondria are incompatible with an AF16 nuclear allele. Two mechanisms potentially cause such a result. First, independent DMIs might have arisen in the mitochondrial and nuclear genomes of each population, such that both cybrid cross directions result in the same phenotypic effect (increased lipid content). This explanation is related to the idea that the existence of multiple DMIs is also predicted to produce more symmetrical effects (Turelli and Moyle 2007). Thus, the presence of a symmetrical effect does not necessarily rule out the existence of DMIs (Turelli and Orr 2000). Second, if mitochondrial–nuclear coevolution has occurred in both populations, then the prediction is that a cybrid resulting from either cross direction would separate that optimal epistatic pair, resulting in a phenotypic effect. For lipid content, we are currently unable to distinguish between the multiple-DMI and the coevolution explanations. Nevertheless, the lipid content data do support the existence of mitochondrial–nuclear epistasis between AF16 and HK104.

### Cybrid fecundity

After observing that mitochondrial physiology is altered in cybrid lines, we sought to identify an organismal phenotype that might correlate mitochondrial function with fitness. The impact of mitochondrial DNA variation on fitness has been recognized (Gemmell *et al.* 2004), and we reasoned that mitochondrial dysfunction might be evident as a decrease in fecundity of cybrids. Other systems with mitonuclear epistasis experience reduced offspring production or sterility, *e.g.*, (Fishman and Willis 2006; Ellison and Burton 2008b). Mitochondrial sequence variation in *C. elegans* is also known to impact sperm motility (Liau *et al.* 2007).

AF16 and HK104 hermaphrodites exhibited indistinguishable self-brood sizes (Figure 5) similar to those previously reported for both strains (Dolgin *et al.* 2007). However, cybrid line fecundities were significantly lower than the paternal parental strain in one cross direction. Thus, the act of exchanging one strain’s mitochondrial haplotype with the other’s has an impact on organismal fitness, and supports the interpretation that mitonuclear epistasis exists in these *C. briggsae* hybrids. The asymmetry seen in the fecundity phenotype suggests the presence of a mitonuclear DMI between the HK104 mitochondrial and AF16 nuclear genomes. It is an interesting correlation that this is the same conclusion reached for ROS levels, although whether a mechanistic connection exists between mitochondrial ROS and fecundity remains to be tested.

### Other potential causes of phenotypic effects in cybrids

In addition to mitochondrial–nuclear effects, it is also important to address whether additional environmental or cytoplasmic factors might explain the phenotypic differences, particularly in fecundity, that we observe between cybrids and their paternal parental strains. With no evidence, to our knowledge, for the existence of Wolbachia or other cytoplasmic parasitic microbes in *C. briggsae*, we must also attempt to rule out other cytoplasmic effects and also potential effects of mitochondrial heteroplasmy.

Given recent reports on the potential impact of maternal effects on reproduction (Harvey and Orbidans 2011; Jobson *et al.* 2015), we were as rigorous as possible about experimentally controlling environmental conditions of individual nematodes involved in phenotyping experiments. We used NGM agar plates containing controlled volumes of agar, and controlled volumes of the same culture of OP50 *E. coli* for nematode husbandry, ensured the presence of OP50 *ad libitum*, maintained all strains in the same incubator, and took particular care to ensure that this rigorous control was extended at least to the parents and grandparents of all phenotyped individuals. This consistency should eliminate the possibility of maternal or even grand-maternal effects on phenotypes.

It has also been noted that naturally occurring heteroplasmy for a mitochondrial genome deletion can occur in *C. briggsae* populations, and that these changes could impact fitness (Howe and Denver 2008). Therefore, we inquired whether heteroplasmy for the *ND5*∆ existed in our cybrid lines, and whether these lines had rapidly evolved different levels of heteroplasmy than the parental strains AF16 and HK104. By our PCR analysis, using primers that distinguish deletion-bearing and intact mitochondrial genomes (Figure 4), no major apparent differences in heteroplasmy levels exist between AF16, HK104, and the five cybrid lines. If any trend exists, digital densitometry suggests that it is a reduction of heteroplasmy in the cybrids. Such a pattern would be predicted, if anything, to improve cybrid fitness.

### Distinguishing DMI from coevolution by effect symmetry

Negative mitochondrial–nuclear epistasis in hybrids might occur in this system by the neutral accumulation of lineage-specific incompatibilities (DMIs), or by selection for compensatory mutation in epistatic pairs (coevolution). It is important to distinguish between these in order to understand the forces that act to generate hybrid dysfunction and ultimately speciation.

Mitochondrial–nuclear epistasis provides a valuable opportunity to more readily distinguish the two possibilities than with nuclear–nuclear epistatic pairs. Under the neutral process by which DMIs accumulate in separate lineages, only one hybrid cross direction should result in the production of dysfunctional hybrids if one of the epistatic loci is in the maternally inherited mitochondrial genome (Turelli and Moyle 2007). Thus, we expect asymmetry in hybrid dysfunction where mitonuclear DMIs exist.

On the other hand, when hybrid dysfunction is due to the separation of coevolved mitochondrial and nuclear loci, one would expect to observe symmetrical effects, because coevolved alleles at two (or more) loci should exist in both parental populations. Thus, any hybrid, regardless of cross direction, should experience the deleterious effects of separating the coevolved alleles of epistatic gene pairs. However, it is also possible that the neutral accumulation of multiple mitonuclear DMIs produces a symmetrical effect (Turelli and Orr 2000; Turelli and Moyle 2007).

Our data reveal asymmetric phenotypic effects in cybrids on fecundity and ROS levels in AF16 (nuclear); HK104 (mt) cybrids. We attribute these effects to the neutral accumulation of mitonuclear DMIs, with the incompatibilities being located in the HK104 mitochondrial and AF16 nuclear genomes. This conclusion can be reconciled with evidence of marker transmission ratio distortion patterns in AF16-HK104 hybrids. We previously found evidence for selection against the combination of AF16 nuclear chromosome V alleles, and HK104 mitotypes, in AI-RIL (Ross *et al.* 2011). This effect is consistent with our interpretation here that ROS levels increase, and fecundity decreases, in cybrids combining the AF16 nuclear and HK104 mitochondrial genomes. However, it remains unclear whether the symmetrical effect of cybridization on lipid content is due to separation of coadapted epistatic pairs, which might provide evidence of the action of selection on the divergence of AF16 and HK104, or whether the symmetry might entirely be due to the neutral independent accumulation of DMIs in both the nuclear and mitochondrial genomes of both AF16 and HK104. In addition to the concept that symmetrical effects could be due to accumulation of multiple independent DMIs (Turelli and Orr 2000; Turelli and Moyle 2007), it has also been noted that such complex incompatibilities are expected to be common (Orr 1995; Coyne and Orr 2004). The presence of DMIs and possibly also coevolved mitochondrial–nuclear loci between AF16 and HK104 suggests that these populations might be experiencing the early stages of speciation.

### Genetic incompatibilities in Caenorhabditis

The first speciation study in Caenorhabditis demonstrated, through attempted hybridization, that *C. elegans* and *C. briggsae* are true biological species (Nigon and Dougherty 1949). Subsequently, mating trials demonstrated that reproductive isolation exists between many pairs of described species of Caenorhabditis (Baird *et al.* 1992; Baird and Yen 2000; Sudhaus and Kiontke 2007; Felix *et al.* 2014). Recent efforts have also probed the basis of interspecies incompatibilities where hybrid offspring are not always completely sterile, such as between *C. briggsae* and its sister species *C. nigoni* (Woodruff *et al.* 2010; Kozlowska *et al.* 2012; Yan *et al.* 2012), and between *C. remanei* and its sister species *C. latens* (Dey *et al.* 2012, 2014).

Fewer cases exist of genetic incompatibility occurring within one Caenorhabditis species. In *C. elegans*, a paternal-zygotic incompatibility involving the *zeel-1* and *peel-1* genes results in embryonic lethality in one interpopulation cross between the Bristol (N2) and Hawaii (CB4856) strains (Seidel *et al.* 2011). Importantly, the *zeel*/*peel* system also displays asymmetry, where embryonic lethality is related to F1 hybrid x P0 cross direction (Seidel *et al.* 2008). Systematic exploration of the landscape of epistatic interactions between these two strains subsequently suggested that multiple deleterious genetic interactions might exist in hybrids (Snoek *et al.* 2014).

We describe here the first naturally occurring intraspecies mitonuclear epistatic interaction in the model genus Caenorhabditis. Our study is the first to create AF16-HK104 cybrids, and the second reported use of cybrids in *C. briggsae* to study the effects of genetic variation on mitochondrial and organismal function (Hicks *et al.* 2012). The identification of a mitonuclear epistatic interaction causing mitochondrial and fitness deficiencies in AF16-HK104 hybrid genotypes represents the third known genetic incompatibility segregating within *C. briggsae* populations. Earlier evidence for potential interstrain genetic incompatibilities showed that AF16-HK104 hybrids produce more embryonic lethality than either parental strain and that ∼20% of the offspring produced by selfing an F1 hybrid take longer to reach adulthood (Dolgin *et al.* 2008). This developmental delay phenotype was later found to be specific to AF16-HK104 hybrids, and to involve a locus on chromosome III (Ross *et al.* 2011; Baird and Stonesifer 2012). Thus, *C. briggsae* is an excellent system in which to study the evolution of genetic incompatibilities within a species.

### Conclusion and future prospects

We provide evidence of neutrally accumulated DMIs that have arisen in two populations of a single species. These DMIs result in asymmetric mitochondrial–nuclear epistatic interactions in *C. briggsae* cybrids. Although producing statistically significant differences in mitochondrial ROS levels and in fecundity, the DMIs only reduce and do not absolutely eliminate hybrid fitness. Additional evidence of incompatibilities segregating within *C. briggsae* comes from the symmetrical effect of mitochondrial–nuclear mismatch on lipid content in hybrids. It is possible that this effect is due to the separation of coevolved mitonuclear epistatic loci.

Although genetic incompatibilities have been found segregating within both *C. elegans* and *C. briggsae*, the phylogeographical population structure present in *C. briggsae* provides a robust framework for testing hypotheses about the evolutionary histories and potentially adaptive values of variants that impact hybrid fitness. Thus, it might not be surprising that the intraspecies *C. briggsae* genetic incompatibilities described thus far exist between a tropical clade strain (AF16) and a temperate clade strain (HK104); most reported incompatibilities do not completely eliminate hybrid fitness. As has been noted, the Dobzhansky-Muller model does not require that the epistatic interaction cause absolute loss of hybrid fitness (*i.e.*, lethality or sterility) (Coyne and Orr 2004). This is arguably a benefit of the *C. briggsae* system and should facilitate more efficient identification of the underlying genetic mechanisms of reduced hybrid compatibility than is possible in systems where hybrids are inviable or infertile. Additional studies will be necessary to investigate the potential adaptive value of mitochondrial divergence and to pursue the possibility that mitochondrial divergence and potential compensatory nuclear mutations might have been driven by ecological forces. Considerable value exists in studying the basis of intraspecific incompatibilities (Maheshwari and Barbash 2011), and *C. briggsae* will continue to be useful for studying the genetic basis of population divergence, the evolution of genetic incompatibilities, and the dynamics of genomic coevolution.
